# A robust ensemble deep learning framework for accurate diagnoses of tuberculosis from chest radiographs

**DOI:** 10.3389/fmed.2024.1391184

**Published:** 2024-07-22

**Authors:** Xin Sun, Zhiheng Xing, Zhen Wan, Wenlong Ding, Li Wang, Lingshan Zhong, Xinran Zhou, Xiu-Jun Gong, Yonghui Li, Xiao-Dong Zhang

**Affiliations:** ^1^Haihe Hospital, Tianjin University, Tianjin, China; ^2^Tianjin Union Medical Center, Nankai University, Tianjin, China; ^3^Tianjin Key Laboratory of Brain Science and Neural Engineering, Academy of Medical Engineering and Translational Medicine, Tianjin University, Tianjin, China; ^4^College of Intelligence and Computing, Tianjin University, Tianjin, China; ^5^Department of Physics and Tianjin Key Laboratory of Low Dimensional Materials Physics and Preparing Technology, School of Sciences, Tianjin University, Tianjin, China

**Keywords:** ensemble deep learning, tuberculosis, chest X-ray radiography, fusion models, clinical screening

## Abstract

**Introduction:**

Tuberculosis (TB) stands as a paramount global health concern, contributing significantly to worldwide mortality rates. Effective containment of TB requires deployment of cost-efficient screening method with limited resources. To enhance the precision of resource allocation in the global fight against TB, this research proposed chest X-ray radiography (CXR) based machine learning screening algorithms with optimization, benchmarking and tuning for the best TB subclassification tasks for clinical application.

**Methods:**

This investigation delves into the development and evaluation of a robust ensemble deep learning framework, comprising 43 distinct models, tailored for the identification of active TB cases and the categorization of their clinical subtypes. The proposed framework is essentially an ensemble model with multiple feature extractors and one of three fusion strategies-voting, attention-based, or concatenation methods-in the fusion stage before a final classification. The comprised de-identified dataset contains records of 915 active TB patients alongside 1,276 healthy controls with subtype-specific information. Thus, the realizations of our framework are capable for diagnosis with subclass identification. The subclass tags include: secondary tuberculosis/tuberculous pleurisy; non-cavity/cavity; secondary tuberculosis only/secondary tuberculosis and tuberculous pleurisy; tuberculous pleurisy only/secondary tuberculosis and tuberculous pleurisy.

**Results:**

Based on the dataset and model selection and tuning, ensemble models show their capability with self-correction capability of subclass identification with rendering robust clinical predictions. The best double-CNN-extractor model with concatenation/attention fusion strategies may potentially be the successful model for subclass tasks in real application. With visualization techniques, in-depth analysis of the ensemble model's performance across different fusion strategies are verified.

**Discussion:**

The findings underscore the potential of such ensemble approaches in augmenting TB diagnostics with subclassification. Even with limited dataset, the self-correction within the ensemble models still guarantees the accuracies to some level for potential clinical decision-making processes in TB management. Ultimately, this study shows a direction for better TB screening in the future TB response strategy.

## 1 Introduction

Tuberculosis (TB) is one of the world's leading infectious disease killers. According to the Global TB Report 2023 released by the World Health Organization, the number of tuberculosis cases worldwide in 2022 was 10.6 million, and the number of deaths was 1.3 million (1). TB remains the world's second leading cause of death from a single source of infection after novel coronavirus infection, causing almost twice as many deaths as human immunodeficiency virus (HIV). Though TB is a serious public health issue, because of its preventable and curable nature, it should be detected and controlled before spreading. Therefore, early screening and diagnosis depend on the accurate identification of TB. Of all the diagnostic methods, chest x-ray radiology (CXR)-based diagnostics are potential candidates for screening TB due to their low cost, fast process, and wide availability. However, CXR image interpretation is limited by the experience and knowledge of radiologists. This difficulty persisted until the advent of remote diagnostics and the development of the Internet and artificial intelligence (AI) in recent years. In current times, more and more CXR images can be transmitted and identified at a low cost and with high accuracy.

The CXR image recognition model has been developed to enrich CXR image datasets in recent decades. Earlier researchers must have collected CXR images and labeled them manually. In 2000, Shiraishi et al. (2) collected 247 chest x-ray cases from 14 medical institutions and built a database to study the pulmonary nodules. The database contains CXR images and associated labels that are labeled by experienced radiologists. According to the Digital Imaging and Communications in Medicine (DICOM) standard introduced by the National Library of Medicine (3), various radiograph images can be collected and distributed among researchers. In 2017, Yu et al. (4) extracted three correlated datasets for lung disease detection from the Japanese Society of Radiological Technology (JSRT) dataset, and a deep learning model was trained to label the “regions of interest” (ROIs) automatically. With the emergence of the CheXpert dataset (5) in 2019, a platform with a collection of numerous CXR images is available worldwide for researchers to model CXR recognition models and algorithms. In 2020, Liu et al. (6) used the TBX11K dataset (including four categories of healthy TB, active TB, latent TB, and unhealthy but non-TB) for image classification and TB region detection for the first time. In 2021, Khatibi et al. (7) proposed a novel multi-instance classification model based on convolutional neural networks and stacked ensemble (CCNSE) using the Montgomery County (MC) dataset and the Shenzhen dataset (SZ).

TB-targeted CXR recognition algorithms have been improved over the past decade. In 2010, Rui et al. (8) proposed a Hybrid Knowledge-Guided (HKG) diagnostic framework for pulmonary TB. The framework contains a mean-shift clustering method of adaptive threshold to detect the object in the ROI, a Gradient Inverse Coefficient of Variation (GICOV) to extract features, and a Bayesian classifier to classify the ROI. In 2015, Hogeweg et al. (9) proposed a computer-aided detection method to identify pulmonary TB based on fusing lung texture, lung shape, and focal features. In 2016, Maduskar et al. (10) proposed an automatic detection method for pleural effusion, which used chest wall contour as a landmark structure to locate the costal diaphragm region, then proposed a region descriptor based on the intensity and shape information of the region around the coastal diaphragm depression, and finally used a random forest classifier to classify the left and right half thorax.

Since the great success of convolutional neural networks (CNN) in the field of image recognition, CNN and its extended algorithms have also been applied to CXR-based TB classifications. In 2017, Lopes and Valiati (11) proposed an ensemble model with pre-trained CNN as a feature extractor and a Support Vector Machine (SVM) classifier. Similar ideas with Multiple Instance Learning (12) as the classifier are reported. In 2017, Paras Lakhani et al. (13) built a similar model that provides medical suggestions to doctors. The CNN model is also used to predict the multi-drug resistance of TB patients (14). In 2020, Ma et al. (15) used a U-Net deep learning algorithm to automatically monitor tuberculosis patients and achieve high accuracy. Feng et al. (16) studied the differential diagnosis of TB granuloma and lung adenocarcinoma. In 2021, Oloko-Oba and Viriri (17) found that five variants of EfficientNets were fine-tuned and implemented on two well-known public CXR datasets (MC and SZ), and EfficientNet-B4 achieved the best accuracy of 92.33% and 94.35% on both datasets. In 2021, Tasci et al. (18), a fine-tuned CNN model based on InceptionV3 and Xception, was used to apply weighted voting and probability mean as combinatorial rules to a soft voting method for two terabytes of CXR image datasets. In 2022, Peng et al. (19) proposed an automatic Hybrid Segmentation Network (H-SegNet) for lung segmentation on CXR. In 2023, Bista et al. (20) used CNNs and YOLO models to detect the consolidation of cavitary patterns of the lesions and their detection. In 2023, Iqbal et al. (21) introduced a novel TB-UNet, which is based on a dilated fusion block (DF) and an attention block (AB) for more accurate segmentation of the lung regions. CNN, which is famous for its feature extraction capability, can be used to extract lesion features and construct deep learning signatures. By addressing biases in CXR recognition tasks through different CNN architectures, ensemble approaches have significantly enhanced the accuracy of image classification tasks.

However, the development of a TB classification model has not yet met the actual needs. One of the problems is the inconsistency/stability of the classification TB model, where a particular model performs significantly better on one CXR dataset than the others (5). Such dataset-targeted performance problems may be linked to overfitting and should be resolved from two aspects: the dataset and the algorithm sides. Another challenge is that clinical diagnosis requires detailed information on the TB subcategory. Therefore, in this study, we propose a new ensemble CNN model that is based on a new training dataset collected from Tianjin Haihe Hospital. In addition, the model incorporates a subclassification algorithm that provides information on TB subclasses. Ensemble CNN models with fine classification for different TB subtypes can be a potential method for clinical diagnostic applications with good consistency.

## 2 Models and dataset for detailed diagnoses

### 2.1 Outline of the dataset

We retrospectively collected 2,191 CXR images (915 TB and 1,276 normal cases) from Tianjin Haihe Hospital, which can enable machine learning models for fine classifications of TB. The images in the dataset have significant characteristics that an experienced doctor can identify. Such manual filtering ensures the quality of images and their tags. Four subsets featuring different characters are constructed, as summarized in Table 1, and the associated statistical profile of the patients can be found in Supplementary Table 1. Thus, there are five classification tasks: normal/TB; secondary tuberculosis/tuberculous pleurisy; non-cavity/cavity; secondary tuberculosis only/secondary tuberculosis and tuberculous pleurisy; and tuberculous pleurisy only/secondary tuberculosis and tuberculous pleurisy. In addition, two public datasets, the SZ and MC CXR datasets, are adopted for consistency testing purposes without subclassification. The results indicate the robustness of both datasets and machine learning models.

**Table 1:** Details of the datasets with extra information on subclasses.

	**Data**	**(From Tianjin Haihe Hospital)**				**Total**
Task1	Normal		1,276			2,191
	TB		915			
Task2	+	Non-cavity	479	Cavity	140	619
Task3	+	Secondary tuberculosis	424	Tuberculous pleurisy	351	775
Task4	+	Secondary tuberculosis only	479	Secondary tuberculosis and tuberculous pleurisy	296	775
Task5	+	Tuberculous pleurisy only	140	Secondary tuberculosis and tuberculous pleurisy	296	436

Details can be used to train the machine learning algorithm for finer diagnoses.

### 2.2 Model

**Figure 1:**
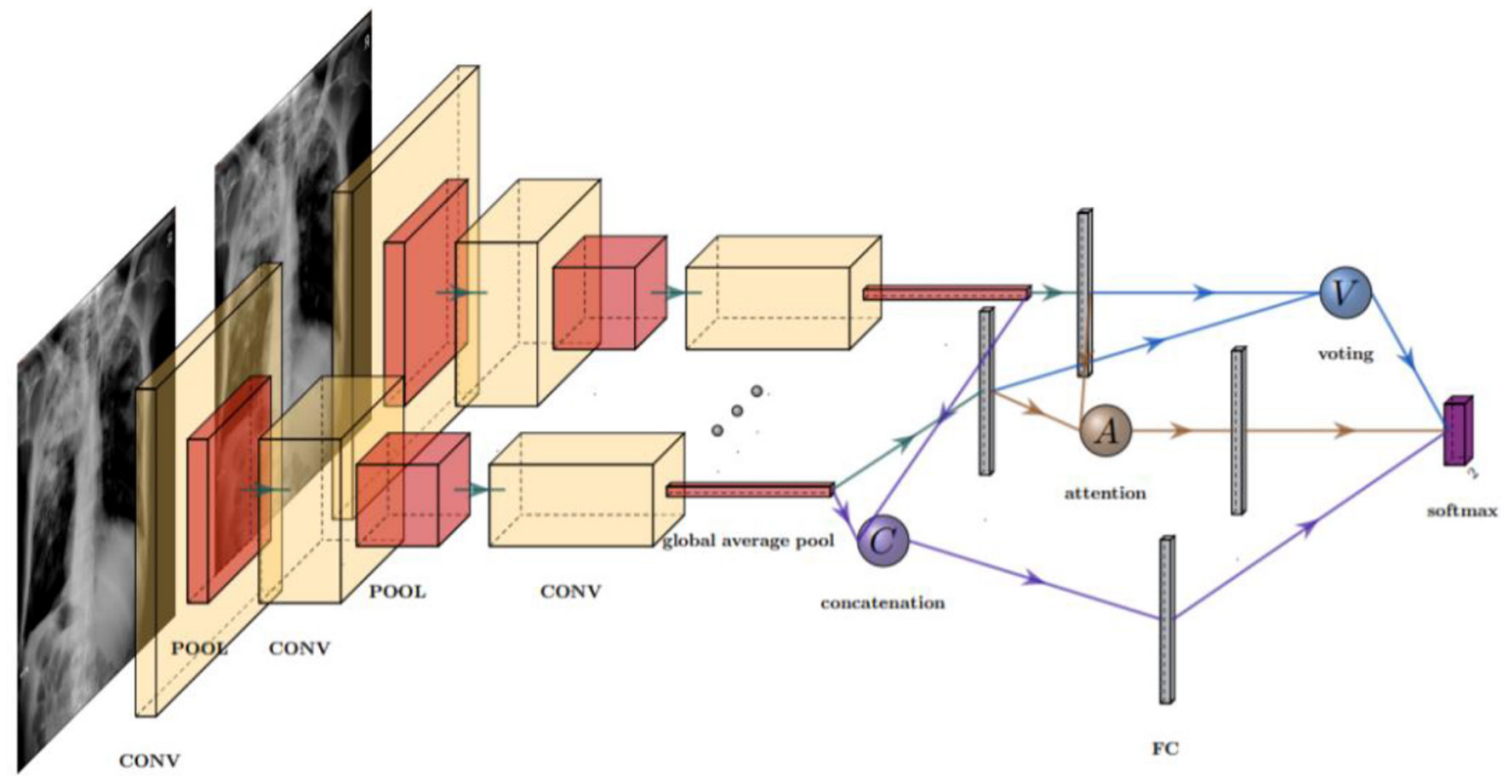
Structure of an ensemble model with three different fusion strategies: concatenation (C), voting (V), and attention (A). In practice, only one fusion strategy is allowed in each model. The fusion layer combines the features extracted by the CNN extractors, which contained several pooling (POOL) and convolution (CONV) layers.

The models we trained in this study take a single-view CXR image as input and output five classification results. The ensemble CNN model structure is shown in Figure 1, which contains one or more feature extractors and a fusion strategy.

#### 2.2.1 Feature extractors

Each feature extractor in this study has been obtained by combining multiple CNN models. The building blocks of a feature extractor are well-performed CNNs that focus on different aspects of images. These CNNs include six classical networks [AlexNet, DenseNet (22), EfficientNet, GoogleNet, MobileNet, and VGGNet (23)]and two brief networks (MtbCNN and MtbNet). In particular, DenseNet121 and DenseNet161 in DenseNet, EfficientNet-es, EfficientNet-b0, EfficientNet-b3, and EfficientNet-b5 in EfficientNet, and VGG11 and VGG13 in VGGNet are selected. MtbCNN and MtbNet refer to a 3-layer and a 5-layer CNN network, which can be easily implemented and trained.

The integration of two compact networks, MtbNet and MtbCNN, enhances the robustness of recognizing simple patterns. Utilizing these models in an ensemble, with one of each type, provides a baseline that demonstrates the effectiveness of classical networks in improving performance. As shall be demonstrated in the “Results” section, only several MtbNet/MtbCNN-containing models have won. The remaining convolution layers are packed into a CNN model to extract features by removing full connection layers in each of the structures. A feature extractor contains 1-3 CNN model(s), which run in parallel and produce features for the next stage. With the 13 basic structures, 142 feature extractors are constructed, including 13 single-CNN extractors, 57 double-CNN extractors, and 72 triple-CNN extractors.

#### 2.2.2 Fusion strategies

Each feature extractor is followed by a fusion phase that merges the extracted features. The feature is collected and merged in the fusion phase based on the high-level feature information obtained from extractors. The fusion strategies that we considered in this research are concatenation, attention, and voting. The concatenation strategy is to pool, flatten, and stack the extracted features into a one-dimensional vector that is passed to the fully connected layer and the softmax layer for classification. The attention strategy calculates only the weighted average of the pooled features and passes the average to the intact connectivity layer for classification. The voting strategy consists of an independent, fully connected layer that acts as a classifier to process each set of extracted features and then averages the predicted output as the final output. The voting strategy considered in this study is essentially a soft voting strategy.

#### 2.2.3 Nomenclature

For simplicity, each model has a unique name, which is defined as


$$\begin{array}{c} \langle fusion\;abbreviations\rangle \langle CNN1\rangle \left[parameter1\right]\left[CNN2\right]\left[parameter2\right]\\ \left[CNN3\;\right]\left[parameter3\right]. \end{array}$$

The abbreviations of fusion strategies and CNNs are summarized in Table 2.

**Table 2:** Abbreviations of the fusion strategies and the CNN names.

**Fusion strategies**								
Abbreviations	Con	Att	Vot	Sig				
Full names	Concatenation	Attention	Voting	Single model without fusion				
**CNN models**								
Abbreviations	Alx	Den	Eff	Ggl	Mbl	Vgg	Mtn	Mtc
Full names	AlexNet	DenseNet	EfficientNet	GoogleNet	MobileNet	VGGNet	MtbNet	MtbCNN
Parameters		121, 161^*^	es, b0, b3, b5^**^			11, 13^***^	

^*^121 and 161 are the number of layers in DenseNet. ^**^es is the lightweight EfficientNet for Google Tensor Processing Unit. b0, b3, and b5 are the compound scaled versions, and the number denotes the scale. ^***^11 and 13 are the years of release of the VGGNet. Hyperparameters can be downloaded as packed pre-trained models in the Pytorch distribution (https://pytorch.org/vision/master/models.html).

#### 2.2.4 Model evaluation

The dataset has been split into training and test sets. A total of 96 CXR images (4%) from the dataset have been reserved for testing in Task1. For the classification of Task2–5, 20% of the CXR images have been retained for testing purposes. Multiple evaluation methods, including accuracy, the area under the Receiver Operating Characteristic (ROC) curve (AUC), the F1 score, sensitivity (sens), and specificity (spec), have been calculated for performance evaluation. For benchmarking purposes, the abovementioned 13 basic structures have also been combined into a hard voting model (voting-13 model) without parameter tuning or other modifications.

#### 2.2.5 Training procedure

Considering the small dataset, we have used a pre-trained model when training the feature extractor for the first time, and a total of 13 different convolutional neural network models have been trained. The ensemble models are trained with the Adam optimizer, cross-entropy loss function, and cosine annealing learning rate adjustment strategy. The batch size and rounds of training are set to 32 and 120. In the training process, the 5-fold cross-validation (CV) method allows parameter tuning with 20% of randomly selected training data. Training for each model is terminated when the score on the CV set does not improve for more than 20 rounds. Then, the model with the best score on the CV set is selected as a feature extractor. Considering that CNNs already have strong feature extraction ability, only fine-tuning of the parameters is allowed by limiting their learning rate to 1/10 of that of the full connection layer. All models have been developed using servers equipped with the NVIDIA GeForce P40 GPU, the Ubuntu system, and Pytorch, a widely used deep-learning framework.

## 3 Results and discussion

### 3.1 Performance of the best ensemble CNN models

The performance results of the best models are summarized in Table 3, with the voting-13 benchmark provided.

**Table 3:** Models with the best performance on datasets.

**Task**		**ACC**	**AUC**	**F1**	**Sens**	**Spec**
1	Best model	VotDen121Effb0	VotDen121Effb0	VotDen121Effb0	VotDen121Effb0	ConAlxDen121
		1	1	1	1	1
	Voting-13	1	1	1	1	1
2	Best model	ConDen161Effb5	AttEffesGgl	AttDen161Effb5	VotAlxDen161	ConEffb0Vgg13
		0.761	0.743	0.698	0.922	1
	Voting-13	0.742	0.712	0.627	0.531	0.89
3	Best model	AttDen161Effb3	AttDen161Effb3	AttDen161Effb3	AttDen161Effb3	ConDen161Vgg13
		0.976	0.984	0.949	1	1
	Voting-13	0.867	0.723	0.619	0.464	0.989
4	Best model	ConAlxDen121	ConAlxDen121	ConAlxDen121	ConDen161Effb3	AttMtnVgg11
		0.865	0.846	0.804	0.8	1
	Voting-13	0.761	0.737	0.661	0.655	0.82
5	Best model	AttDen161Effb5	AttDen161Effb5	ConDen121Effb5Ggl	AttMtnVgg13	AttAlxDen161Effb5
		0.805	0.805	0.862	1	0.84
	Voting-13	0.747	0.655	0.831	0.871	0.44

The performance of the voting-13 model is provided as a benchmark. The performance indicates that the number of feature extractors must be limited for the fusion to take effect.

Task1, TB identification is the easiest classification test. The hard voting-13 algorithm and many ensemble models can achieve the best performance. The dataset with a total of 2,191 CXR images is sufficient to train CNN models, and the pathological features of CXR in TB patients are prominent. In addition, according to the Vapnik-Chervonenko-dimension theory (VC dimension theory), a simpler machine learning system is always preferred to a complicated one to avoid over-fitting issues. Therefore, simpler CNNs such as Efficientb0 or Efficientb3 are the best ones to provide the desired accuracy for Task1.

Task2–5 are more difficult than Task1 because of the smaller sample sizes and ambiguity in the CXR images. In Task2, the best ensemble models outperform the benchmarks by only a small amount. Since cavities are similar to small bubbles, which are commonly observed in lung images, misclassification may happen when CNNs extract small bubbles as cavities. Ensemble model ConDen161Effb5 achieves the best ACC (0.761), while ensemble model AttEffesGgl achieves the best AUC (0.743). Task3–5 are easier for the ensemble models, and performance improvements are significant. Ensemble models achieve ACC = 0.976 and AUC = 0.984 in Task3, ACC = 0.865 and AUC = 0.846 in Task4, ACC = 0.805 and AUC = 0.805 in Task5, respectively.

In practice, specificity is the most important index in medical applications and should be used for model evaluation purposes. Since specificity is defined as the ratio of the true negative and the total recognized negative to be used for medical screening to exclude negative samples, in this context, the best models for Task1–5 are ConAlxDen121, ConEffb0Vgg13, ConDen161Vgg13, AttMtnVgg11, and AttAlxDen161Effb5.

We have compared the best-performing methods in Task1–5 with those already used in some of the current studies. Table 4 shows this study's efficiency by collecting the selected study's accuracy and then comparing it to the accuracy of this study.

**Table 4:** The accuracy of the proposed method in comparing the existing studies to classify normal vs. TB.

**Approaches**	**Authors**	**Classes**	**Accuracy %**	**References**
ResNet50	Wang et al.	Normal vs. TB	82.2	(24)
EfficientNet	Tan and Le	Normal vs. TB	83.3	(25)
FuseNet	Abdar et al.	Normal vs. TB	91.6	(26)
MAG-SD	Wang et al.	Normal vs. TB	96.1	(27)
CoroNet	Khan et al.	Normal vs. TB	93.6	(28)
TBXNet	Ahmed et al.	Normal vs. TB	98.9	(29)
Ensemble CNN	Proposed method	Normal vs. TB	100.0 (SZ:98.2 MC:99.3)^*^	This work

Detailed classification performances from previous reports are not available. ^*^The accuracy values are not validated across different datasets.

### 3.2 Best-performing models and their characteristics

When the best model is selected (Table 4), the double-CNN-extractor models dominate the set of best models. Since soft voting considers the extracted features in a single vector representation before the average, one can consider voting as a simpler fusion. In contrast, concatenation can be considered as a “static complex” fusion, whereas attention can be considered as a “dynamic complex” fusion. As shown in Table 5, a soft fusion may be the best approach in Task1 when there is no high requirement for specificity. In contrast, complex fusion strategies appear more frequently than voting in cases with low data supply since combining extracted features with a non-linear algorithm may be a better choice than a single-valued vote. However, when the data are sufficiently large, CNN models should be evaluated independently before the fusion step is taken.

**Table 5:** The best model in single-, double-, and triple-CNN-extractor model sets.

**Task**		**ACC**	**AUC**	**F1**	**Sens**	**Spec**
1	Single	SinEffb0	SinEffb0	SinEffb0	SinEffb0	SinEffb161
	Double	VotDen121Effb0	VotDen121Effb0	VotDen121Effb0	VotDen121Effb0	ConAlxDen121
	Triple	VotDen121Effb0Vgg13	VotDen121Effb0Vgg13	VotDen121Effb0Vgg13	VotDen121Effb0Vgg13	ConAlxDen121Effb0
2	Single	SinDen121	SinDen121	SinDen161	SinDen161	SinAlx
	Double	ConDen161Effb5	AttEffesGgl	AttDen161Effb5	VotAlxDen161	ConEffb0Vgg13
	Triple	ConDen161Effb0Ggl	VotMblDen161Effb0	VotMblDen161Effb0	VotAexnetDen161Effb0	AttAlxDen161Effb3
3	Single	SinDen161	SinDen161	SinDen161	SinDen161	SinAlx
	Double	AttDen161Effb3	AttDen161Effb3	AttDen161Effb3	AttDen161Effb3	ConDen161Vgg13
	Triple	ConDen161Effb3Ggl	ConDen161Effb3Ggl	ConDen161Effb3Ggl	ConDen161Effb3Ggl	AttDen161Effb3Vgg13
4	Single	SinDen161	SinDen161	SinDen161	SinDen161	SinVgg13
	Double	ConAlxDen121	ConAlxDen121	ConAlxDen121	ConDen161Effb3	AttMtnVgg11
	Triple	ConDen121Effb5Ggl	ConDen121Effb5Ggl	ConDen121Effb5Ggl	ConDen121Effb5Ggl	ConMtcMtnVgg13
5	Single	SinDen161	SinGgl	SinDen161	SinDen161	SinGgl
	Double	AttDen161Effb5	AttDen161Effb5	AttDen161Effb5	AttMtnVgg13	AttDen161Effb5
	Triple	ConDen121Effb5Ggl	ConDen161Effb3Ggl	ConDen121Effb5Ggl	ConMtcMtnVgg13	AttDen121Effb3Ggl

### 3.3 Self-correction in the ensemble framework

**Figure 2:**
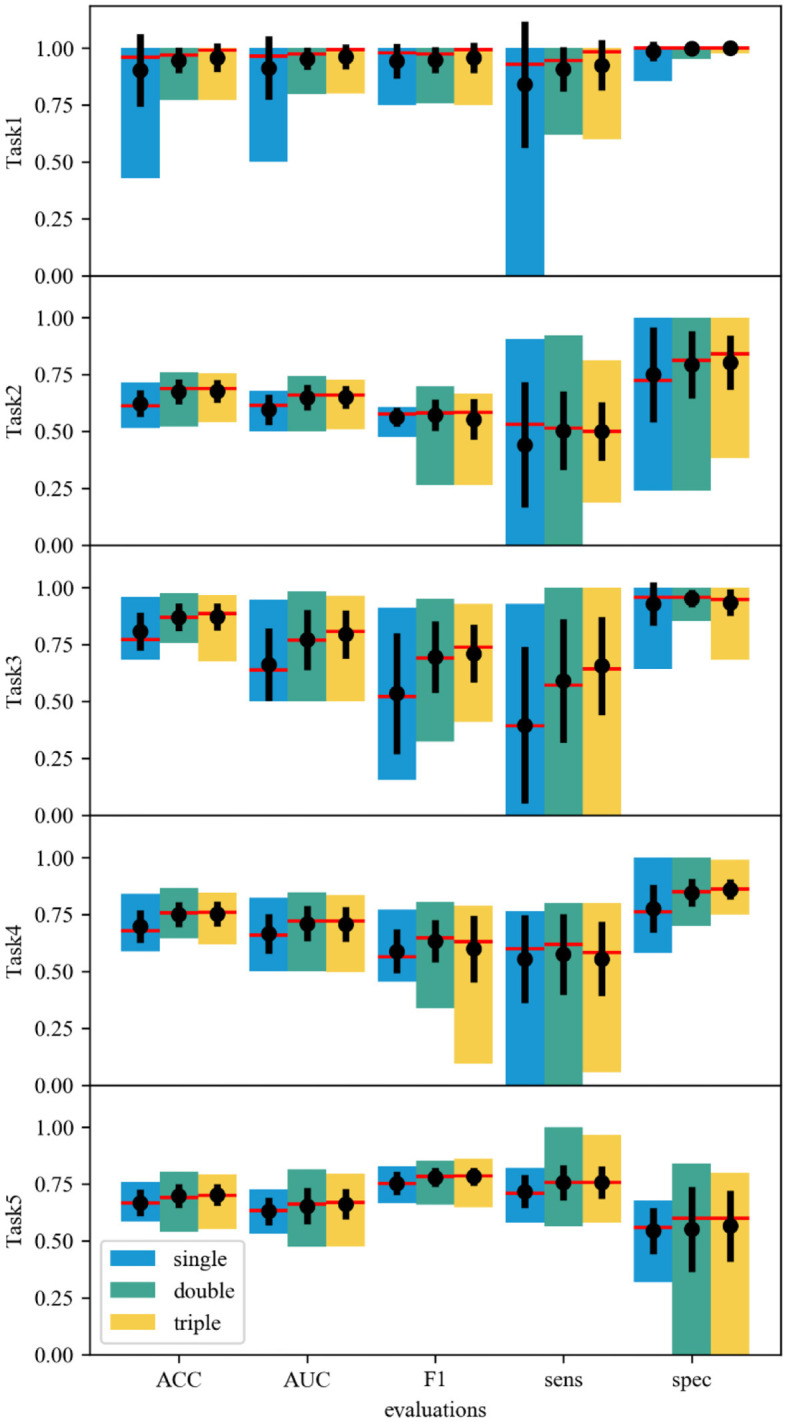
Statistical data of different ensemble models. The single-, double- and triple-CNN model sets correspond to the blue, cyan, and yellow bars. The top and bottom of each bar correspond to the maximum and minimum values. A red line across each bar denotes the median value. The central black dot with an error line in each bar shows the mean value and the standard deviation.

The power of the ensemble framework relies on the self-correction in the fusion part, which allows the misclassified features to be adjusted by another extractor(s). To represent the “self-correction” impacts of the ensemble framework, statistics with respect to the model variant (single, double, and triple CNN structures) are focused, as shown in Figure 2. Based on the statistics across models of a set of 13 single-CNN extractors, a set of 57 double-CNN extractors, and a set of 72 triple-CNN extractors, the medians and means are improved as the number of CNN extractors increases in all evaluation aspects, including ACC, AUC, F1, sens, and spec, despite few exceptions. The results show the emergence of self-correction in most indexes of all five classification tasks. For example, regarding the AUC and ACC statistics, the medians (red lines) and the means (black dots) are generally improved with the increase of extractor numbers.

Besides the improvement of medians and means, models' performances are polarized, which can be associated with large standard deviations in some cases. Concretely, the self-correction improves the F1 score on average, but it also gives the models a much larger standard deviation in some cases, such as Task4. The only reduction in sensitivity is noticed in Task2, where more CNN extractors worsen the results. In tasks where high sensitivity is needed, a detailed evaluation must be considered. As a result, a good model must be selected with careful evaluation. The results suggest that the marginal gain from increasing the number of CNN extractors drops significantly. Thus, limiting the ensemble model to double-CNN-extractor models may be the best practice.

In addition, the double- and triple-CNN model error bars are comparable in most cases. The statistical data also points out the skewness of the model sets. Most model sets show skewness to the high score side, which indicates that most models can capture the features correctly. The performance is further enhanced when multiple CNN extractors are allowed in one model. In the ACC scores of Task2–5, the skewness to the low score side in these model sets can be improved by adding 1 or 2 CNN extractors. Multiple CNN extractors can correct some misbehavior of a single CNN extractor. Even in Task2, which has a low data supply, the improvement in the skewness can still be observed.

### 3.4 Independent validation of public CXR datasets

Independent validation tests are performed with the SZ and MC CXR image datasets. Based on the previous discussions, double-CNN-extractor models are focused, and single-CNN-extractor models are used as benchmark models. After 19 experiments, the results indicate that the best double-CNN-extractor model performs better than the best single-CNN-extractor model, as shown in Table 6. The double-CNN-extractor model shows its potential across various datasets. The best double-CNN-extractor model improves the ACC score by 1%−10%. Such dataset dependency may be improved by incorporating a larger dataset in the future.

**Table 6:** Independent validation results of the best single- and double-CNN-extractor models trained with the Haihe, SZ, and MC TB datasets.

**The best double-CNN-extractor model**				**The best single-CNN-extractor model**			
	**Haihe**	**SZ**	**MC**		**Haihe**	**SZ**	**MC**
Haihe	**1**	0.651	0.5	Haihe	**1**	0.556	0.58
SZ	0.625	**0.982**	0.623	SZ	0.573	**0.977**	0.616
MC	0.698	0.711	**0.993**	MC	0.594	0.687	**0.971**

The best double-CNN-extractor model performs significantly better than the best single-CNN-extractor model. The first cell of each row represents the dataset used to train the model. Bold values represent that the models are trained and tested with the same dataset.

Table 7 shows the detailed statistics of independent tests. The models appear to be dataset dependent, showing low scores when validations are performed across the dataset. The first row of data in each cell represents the test results of a single model, and the second row represents the statistics among all double-CNN-extractor models. The 3–5 rows of data are the results of concatenation, attention, and voting strategies, respectively. The poor performance of non-diagonal cells represents the limitations of datasets. This limitation could be traced to the divergence of datasets, which may have originated from the year gaps, the equipment differences, or other factors. However, based on the current datasets, one can achieve a fair-to-good performance by selecting the MC dataset as the training set with the attention fusion strategy and double-CNN-extractors. After careful benchmarking, a maximum ACC of 0.698 can be expected. Such an approach may have the best chance of targeting TB diagnosis in a new environment. As a result, merging different datasets into a single model may not be an appropriate approach because of the inner bias in every dataset.

**Table 7:** Independent validation results of the best single- and double-CNN-extractor models trained with the Haihe, SZ, and MC TB datasets.

	**Haihe**	**SZ**	**MC**
Haihe	0.901, 0.427, 0.958, 1.0	0.494, 0.382, 0.508, 0.556	0.439, 0.42, 0.42, 0.58
	0.945, 0.771, 0.969, 1.0	0.5, 0.361, 0.506, 0.651	0.424, 0.391, 0.42, 0.5
	0.924, 0.771, 0.969, 1.0	0.481, 0.361, 0.505, 0.529	0.423, 0.413, 0.42, 0.435
	0.941, 0.823, 0.958, 1.0	0.518, 0.403, 0.508, 0.651	0.428, 0.391, 0.42, 0.5
	0.97, 0.906, 0.969, 1.0	0.5, 0.453, 0.503, 0.562	0.421, 0.42, 0.42, 0.428
SZ	0.49, 0.333, 0.49, 0.573	0.888, 0.508, 0.956, 0.977	0.529, 0.42, 0.543, 0.616
	0.453, 0.292, 0.448, 0.625	0.901, 0.802, 0.881, 0.982	0.554, 0.399, 0.565, 0.623
	0.439, 0.354, 0.427, 0.542	0.871, 0.811, 0.869, 0.964	0.547, 0.399, 0.565, 0.58
	0.43, 0.292, 0.427, 0.625	0.864, 0.802, 0.869, 0.964	0.536, 0.42, 0.551, 0.58
	0.489, 0.406, 0.479, 0.583	0.968, 0.94, 0.97, 0.982	0.578, 0.522, 0.587, 0.623
MC	0.506, 0.333, 0.521, 0.594	0.517, 0.376, 0.508, 0.687	0.861, 0.58, 0.942, 0.971
	0.54, 0.406, 0.552, 0.698	0.531, 0.423, 0.518, 0.711	0.889, 0.42, 0.949, 0.993
	0.535, 0.427, 0.531, 0.656	0.51, 0.458, 0.506, 0.588	0.902, 0.58, 0.964, 0.993
	0.55, 0.427, 0.552, 0.698	0.531, 0.423, 0.518, 0.711	0.823, 0.42, 0.935, 0.986
	0.536, 0.406, 0.562, 0.573	0.534, 0.498, 0.517, 0.687	0.942, 0.891, 0.949, 0.978

There are five rows in each cell (validation). The five rows are statistics of single-CNN-extractor models, all double-CNN-extractor models, double-CNN-extractor models with concatenation fusion, double-CNN-extractor models with attention fusion, and double-CNN-extractor models with voting fusion. The four values in each cell's row are the mean, the minimum, the median, and the maximum. The hanging cell in each row represents the dataset used to train the model.

### 3.5 The impact of different fusion strategies

Finally, the self-correction can be further displayed with Gradient-weighted Class Activation Mapping (GradCAM) (30) visualizations. GradCAM is a visualization technique that utilizes the backpropagation gradient information of a convolutional neural network to highlight the regions in the input image that contribute the most to the prediction of a particular class. Based on the extracted features and back-propagated gradients, the two visualization algorithms can highlight critical regions of CXR images for model reasoning. The fusing in the ensemble model provides further resolution and correction to the images.

**Figure 3:**
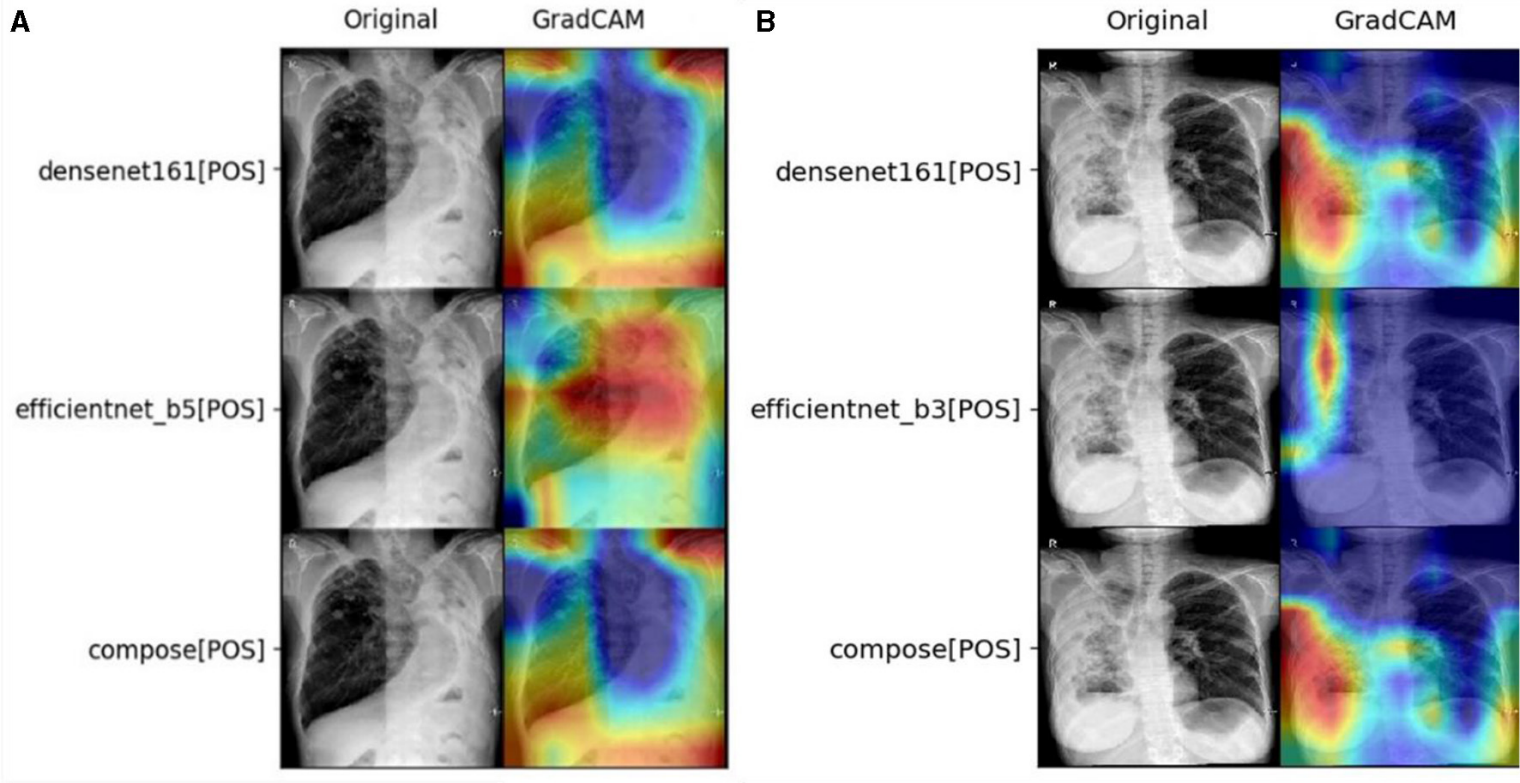
Demonstration of self-correction in the fusion layer for **(A)** Task2 with the ACC champion, ConDen161Effb5, and **(B)** Task4 with the sens champion, ConDen161Effb3.

As demonstrated in the statistical data, the self-correction should be observed as the win of a good feature extractor at the fusing stage. Taking Task2 as an example, the prediction effect of the fused double CNN models Den161 and Effb5 has achieved good results in ACC (0.761) and F1 (0.698), as shown in Figure 3A. In this case, Dens161 corrects Effb5 with its correct focus on the left side of the lung. In addition, as demonstrated in Figure 3B, Den161 corrects Effb3 by enlarging the focus of the left lung. Such behavior explains the origin of the high sensitivity, which can be linked to the full coverage of TB regions. Such correct behavior is the key for such an algorithm to win the sensitivity of double-CNN-extractor models in Task4.

**Figure 4:**
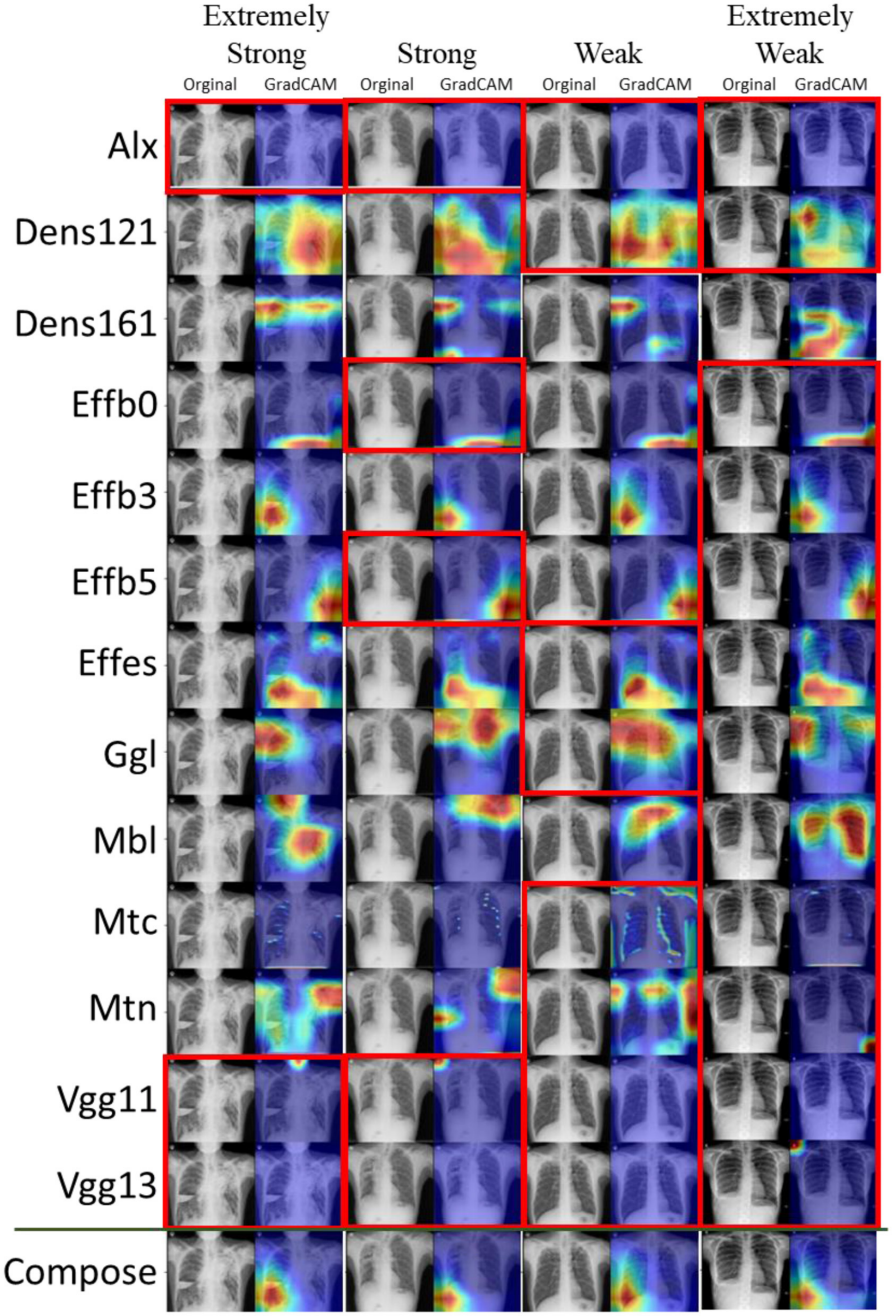
Task2: Voting-13 model with feature visualization of the 13 basic CNN extractors and the voting layer. Four panels from left to right correspond to an extremely strong, a strong, a weak, and an extremely weak case of prediction. Negative predictions are marked by red boxes. When reading columnwise, one can see the voting procedure for the ensemble model.

To obtain insights into the 13 basic CNN structures and how hard voting works, the results of the voting-13 models for Task2 are shown in Figure 4. Moreover, four typical examples are selected to represent extremely strong positive (only 3 negative votes), strong positive (5 negative votes), weak positive (9 negative votes), and extremely weak positive (12 negative votes) cases. The correction of voting is not just voting in common sense but a further process by a fully connected layer. Therefore, a more positive count does not mean that the fused result turns positive. The fully connected layer may selectively synthesize results with weights. However, such fusion may easily bring more bias to the fusion. Thus, the overall performance of the voting-13 model in Task2 is poorer than its performance in Task1. When a few CNN extractors extract key features correctly, wrong predictions are usually made. Thus, it is critical to control the scale of the feature extractor to a small number.

In the best double-CNN-extractor models, statistical results indicate that the results obtained by the fusion strategy are better than those obtained by hard voting. In these ensemble models, the fusion strategy can effectively utilize the key features obtained by the feature extractor. Whether the model can distinguish the key positive and negative features is particularly important.

## 4 Summary

In this study, ensemble models with CNN-extractors and fusion layers are proposed and applied to the Haihe TB CXR image dataset for five classification tasks. With the extracted features from CNN extractors, three fusion strategies are explored: concatenation, voting, and attention. With the limitations of the data, the ensemble models can improve the accuracy of TB classification tasks. The performance of single-, double-, and triple-CNN-extractor models is extensively discussed and compared. Double-CNN-extractor models can be promising model candidates in multi-purpose CXR image classifications with a smaller risk of overfitting. The best double-CNN-extractor models are ConAlxDen121 (for Task1), AttDen161Effb3 (for Task2), AttAlxDen161Effb5 (for Task3), ConDen161Effb3 (for Task4), and ConDen121Effb5Ggl (for Task5). Besides, using voting as a fusion is a simple but effective approach. If one limits the number of feature extractors to a small number, it is suitable for simple tasks, and attention and concatenation strategies are more reliable when the task is complex. An attention strategy is more suitable for double CNN models due to its weighting characteristics, and the concatenation strategy is more suitable for fused triple CNN models with complex and fine tasks due to its characteristics of retaining information integrity. For different types of medical imaging tasks, choosing the appropriate model and fusion strategy can improve the prediction effect, compensate for the shortcomings of a single model to a certain extent, and provide more reliable support for medical imaging diagnosis.

However, there are still some challenges in classifying cavities, particularly because small cavities and bubbles in CXR images often appear very similar, especially given the low resolution of these images. This similarity can lead CNN extractors to misidentify features, resulting in incorrect predictions. Future research should concentrate on refining model behavior to address these issues more accurately.

## Data availability statement

The datasets presented in this article are not readily available because dataset contains 2191 X-ray images collected within from Haihe Hospital premises that cannot be made a public dataset. Requests to access the datasets should be directed to ZW, 18920180078@189.cn.

## Ethics statement

The studies involving humans were approved by ZX, Tianjin Haihe Hospital. The studies were conducted in accordance with the local legislation and institutional requirements. The participants provided their written informed consent to participate in this study.

## Author contributions

XS: Data curation, Investigation, Resources, Writing – original draft, Methodology, Supervision. ZX: Conceptualization, Formal analysis, Writing – original draft, Data curation, Resources. ZW: Writing – original draft, Methodology. WD: Data curation, Writing – original draft. LW: Formal Analysis, Writing – original draft. LZ: Writing – original draft, Conceptualization. XZ: Visualization, Writing – original draft. X-JG: Writing – original draft, Software, Validation, Visualization. YL: Writing – original draft, Conceptualization, Formal analysis. X-DZ: Funding acquisition, Project administration, Supervision, Validation, Writing – original draft.
