# Supplementary material for: A robust ensemble deep learning framework for accurate diagnoses of tuberculosis from chest radiographs
Source: Front Med (Lausanne). 2024 Jul 22;11:1391184. doi: 10.3389/fmed.2024.1391184 (PMC11301748; doi:10.3389/fmed.2024.1391184)
Supplement: Supplementary file 1 [file Table_1.pdf]

Statistics of Haihe CXR image datasets

Supplementary Table 1 provides a brief statistic of Haihe CXR image datasets.

Characteristics	TB	Normal
Overall	915	1276
Age (mean/std deviation)	44.17±18.96	34.81±11.37
(median/IQR)	42 (27-59)	32 (26-42)
Gender		
male	675 (74%)	872 (68%)
female	240 (26%)	404 (32%)

Supplementary Table 1. Statistics of the characteristics of participants.
